# A Motion Capture Study to Measure the Feeling of Synchrony in Romantic Couples and in Professional Musicians

**DOI:** 10.3389/fpsyg.2016.01673

**Published:** 2016-10-27

**Authors:** Delphine Preissmann, Caecilia Charbonnier, Sylvain Chagué, Jean-Philippe Antonietti, Joan Llobera, Francois Ansermet, Pierre J. Magistretti

**Affiliations:** ^1^Agalma FoundationGeneva, Switzerland; ^2^Department of Child and Adolescent Psychiatry, Faculty of Medicine, University of Geneva HospitalsGeneva, Switzerland; ^3^Cognitive Science Center, University of NeuchâtelNeuchâtel, Switzerland; ^4^Medical Research Department, Artanim FoundationGeneva, Switzerland; ^5^Institute of Psychology, University of LausanneLausanne, Switzerland; ^6^Immersive Interaction Group, Ecole Polytechnique Fédérale de LausanneLausanne, Switzerland; ^7^Division of Biological and Environmental Sciences and Engineering, King Abdullah University of Science and TechnologyThuwal, Saudi Arabia; ^8^Brain Mind Institute, École Polytechnique Fédérale de LausanneLausanne, Switzerland

**Keywords:** synchrony, mirror game, motion capture, musicians, subjective feeling, quality of interactions

## Abstract

The feeling of synchrony is fundamental for most social activities and prosocial behaviors. However, little is known about the behavioral correlates of this feeling and its modulation by intergroup differences. We previously showed that the subjective feeling of synchrony in subjects involved in a mirror imitation task was modulated by objective behavioral measures, as well as contextual factors such as task difficulty and duration of the task performance. In the present study, we extended our methodology to investigate possible interindividual differences. We hypothesized that being in a romantic relationship or being a professional musician can modulate both implicit and explicit synchronization and the feeling of synchrony as well as the ability to detect synchrony from a third person perspective. Contrary to our hypothesis, we did not find significant differences between people in a romantic relationship and control subjects. However, we observed differences between musicians and control subjects. For the implicit synchrony (spontaneous synchronization during walking), the results revealed that musicians that had never met before spontaneously synchronized their movements earlier among themselves than control subjects, but not better than people sharing a romantic relationship. Moreover, in explicit behavioral synchronization tasks (mirror game), musicians reported earlier feeling of synchrony and had less speed errors than control subjects. This was in interaction with tasks difficulty as these differences appeared only in tasks with intermediate difficulty. Finally, when subjects had to judge synchrony from a third person perspective, musicians had a better performance to identify if they were present or not in the videos. Taken together, our results suggest that being a professional musician can play a role in the feeling of synchrony and its underlying mechanisms.

## Introduction

Mimicry and behavioral synchrony play a fundamental role in social interactions by promoting prosocial behavior (van Baaren et al., 2004). Synchrony of body movements, like finger tapping movements (Repp, 2005; Hove and Risen, 2009) or walking in synchrony (Wiltermuth and Heath, 2009) increases affiliation and cooperation. For instance, body synchrony is involved in psychotherapy as it increases the outcome of the psychotherapy session (Ramseyer and Tschacher, 2011, 2014). Prosocial effects of behavioral synchronization are also present in infants (Carpenter et al., 2013) suggesting that it involves fundamental social function appearing early in development. For example, very young infants that were bounced in synchrony with an adult, later show more altruistic behavior toward this adult (Trainor and Cirelli, 2015). Recently, interpersonal synchrony feelings have also been linked to physiological markers like heart rate in couples performing an action imitation game (Noy et al., 2015). Mimicry and behavioral synchrony can be spontaneous and non-conscious or can be explicit like in joint action, but the precise relations between implicit and explicit mimicry are still unknown (see for a review Sebanz et al., 2006; Knoblich et al., 2011). Both explicit and implicit mimicry might involve a prediction of others' action outcomes relying on neuronal mirror system and are fundamental for intersubjectivity (Gallese, 2003; Iacoboni et al., 2005; Sebanz et al., 2006). Behavioral synchrony increases cooperation by mechanisms that might involve shared intentionality (Reddish et al., 2013). The relation between behavioral imitation and bonding is 2-fold. On one hand, we like more people that mimic our behavior (Chartrand and Bargh, 1999). On the other hand, we mimic more people that we like (Stel et al., 2010) and prosocial personalities have a greater tendency to synchronize their body movements than pro-self-oriented individuals (Lumsden et al., 2012).

Synchrony can thus be affected by the relationship between people. Indeed, previous studies have shown that people in a romantic relationship unconsciously mimic less an attractive sex opposite person, presumably to protect their present romantic relationship from potential attractive alternatives (Karremans and Verwijmeren, 2008), suggesting that behavioral synchrony is a fundamental aspect in romantic relationships. Moreover, previous studies suggest that moving in synchrony increases cooperation even if it has a negative impact on the individual (Wiltermuth and Heath, 2009), which might be very important in a relationship.

Interestingly, the feeling of synchrony can also be increased by music (Demos et al., 2012) and prosocial effect of synchrony can be favored by music in infants (Kirschner and Tomasello, 2010). Music has been proposed to have an important function in social bounding (Hagen and Bryant, 2003; Clayton, 2009) possibly by the synchrony it provokes in groups of individuals (Wiltermuth and Heath, 2009) but the mechanisms underlying this effect are not precisely known. Moreover, dancers and musicians often report special moment of feeling of synchrony during improvisation (Hart et al., 2014). This suggests that musicians, in reason of their training, should be better at behavioral synchronization and more sensitive to it (i.e., a stronger relation between behavioral synchronization and subjective feeling of synchrony).

The relation between behavioral synchrony and the feeling of synchrony is still not precisely known. A previous study suggested that the subjective feeling of synchrony is affected by behavioral synchrony, as reflected in measures extracted from objective motion data and, possibly, by psychological factors such as empathy or emotions (Llobera et al., 2016). Moreover, if there are reasons to hypothesize that the feeling of synchrony and behavioral synchronization might be affected by intergroup differences (e.g., being a professional musician or being in a romantic relationship) there have been very few studies investigating these possible differences. To our knowledge, no study investigated how these possible intergroup differences in subjective feeling of synchrony might be sustained by objective difference in behavioral synchronization assessed for instance by motion capture.

Thus, in the present study, the methodology of (Llobera et al., 2016) was extended to investigate possible interindividual differences for subjective feeling in subjects (1) *sharing a romantic relationship* and (2) *being a professional musician* in relationship with objective measures of behavioral synchronization assessed by motion capture. We hypothesized that behavioral synchronization and the feeling of synchrony should appear earlier in musicians and people in a romantic relationship than control subjects. Moreover, we also hypothesized that musicians or people in a romantic relationship might also have better performance in a third person perspective task to detect synchrony and their presence in the interactions.

## Methods

### Participants

All participants provided written informed consent after receiving a detailed explanation of the experimental procedure. They received a small amount of money for their participation (40 Swiss Francs). The Institutional Review Board of the University Hospitals of Geneva approved all experimental procedures for this study. Participants were excluded if they had a history of neurological or mental disorder such as seizure, stroke, mood disorder or depression.

*Couples in a romantic relationship:* A total of 20 subjects (10 females, 10 males, mean ± standard deviation (SD) age: 36.8 ± 11.9 years) were included. The sole exclusion criteria was whether they were not in stable relationship for at least 1 year.

*Musicians:* A total of 12 professional musicians (6 females, 6 males, mean ± SD age: 34.2 ± 10.7 years) participated to the study. They had all been playing in a music group for more than 11 years and practiced for more than 15 h per week.

In the present study, the performance of the two aforementioned groups were compared to a group of an earlier study (Llobera et al., 2016) acting as a *control group* (10 males, 10 females, mean ± SD age: 33.8 ± 10.7 years). Both the musicians and the subjects of the control group were randomly assigned an unknown partner to form a male-female dyad.

### Material, design and procedure

The material, design and procedure has been described in detail elsewhere (Llobera et al., 2016). For the comprehension of the reader, the protocol can be summarized as follows:

The experiment was divided into two parts: a motion experiment and a video test session conducted 2 months later. Upon arrival the two participants completed questionnaires to assess subjective factors before the movement tasks: an empathy questionnaire (Davis, 1980) and the Positive and Negative Affect Schedule (PANAS) questionnaire (Watson et al., 1988; Gaudreau et al., 2006) as a state measure of positive and negative affect. The PANAS questionnaires were also filled in in the middle of the experiment and after the motion tasks to assess possible effects of the tasks on positive and negative affect.

In addition, the couples in a romantic relationship had to fill in two additional questionnaires. First, a revised and abbreviated form of the Dyadic Adjustment Scale (DAS) (Spanier, 1976; Antoine et al., 2008). This questionnaire assesses two different dimensions: the degree of agreement (DA) and the quality of the dyadic interactions (IQ) (Antoine et al., 2008). Second, the Relationship Scales Questionnaire (RSQ, Griffin and Bartholomew, 1994) which assesses self-reported adult attachment.

#### Motion experiment

**Figure 1:**
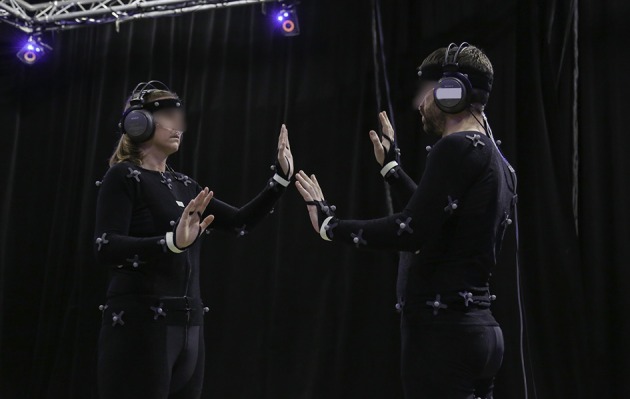
**Experimental setup during the mirror game**. One can see the two participants equipped with reflective markers, phosphorescent tapes, a thermistor-based SleepSense Flow sensor and headphones. For illustration reason, the light is on, but the tests were performed in the dark.

Motion data was captured using a Vicon MXT40S motion capture system (Vicon, Oxford Metrics, UK) consisting of ten infrared cameras, sampling at 120 Hz. Participants were equipped with a Velcro^TM^ motion capture suit and with 53 reflective markers placed on each joint to track the full body motion during the experiment (Figure 1).

##### Implicit synchronization

Participants were first instructed to walk in a circle indicated on the floor in the measurement volume, both in the same direction at a self-selected speed and starting at opposite sides of the circle. They were unaware that the objective of this task was to measure their ability to spontaneously synchronize during walking (we used a cover story as participants were told that they had to walk for the calibration of the motion capture system). Participants were asked to walk twice in clockwise and counterclockwise directions in a counterbalanced manner between dyads. The experimenters counted seven laps before stopping each walk.

##### Explicit synchronization

Next, the participants were equipped with headphones playing continuous white noise to isolate them from ambient noise and verbal cues. They were also equipped with a thermistor-based SleepSense Flow sensor placed beneath the nose, collecting respiration data at 256 samples per second. The sensor was connected to a biosignal amplifier (g.USBamp, g.tec, Schiedlberg, Austria) which had a 30.0 Hz low-pass filter and a 0.1 Hz high-pass filter, as well as a 50 Hz notch filter to suppress the power line interference. A specific trigger procedure (Llobera et al., 2016) was implemented to ensure synchronization between the motion capture and respiration data.

**Figure 2:**
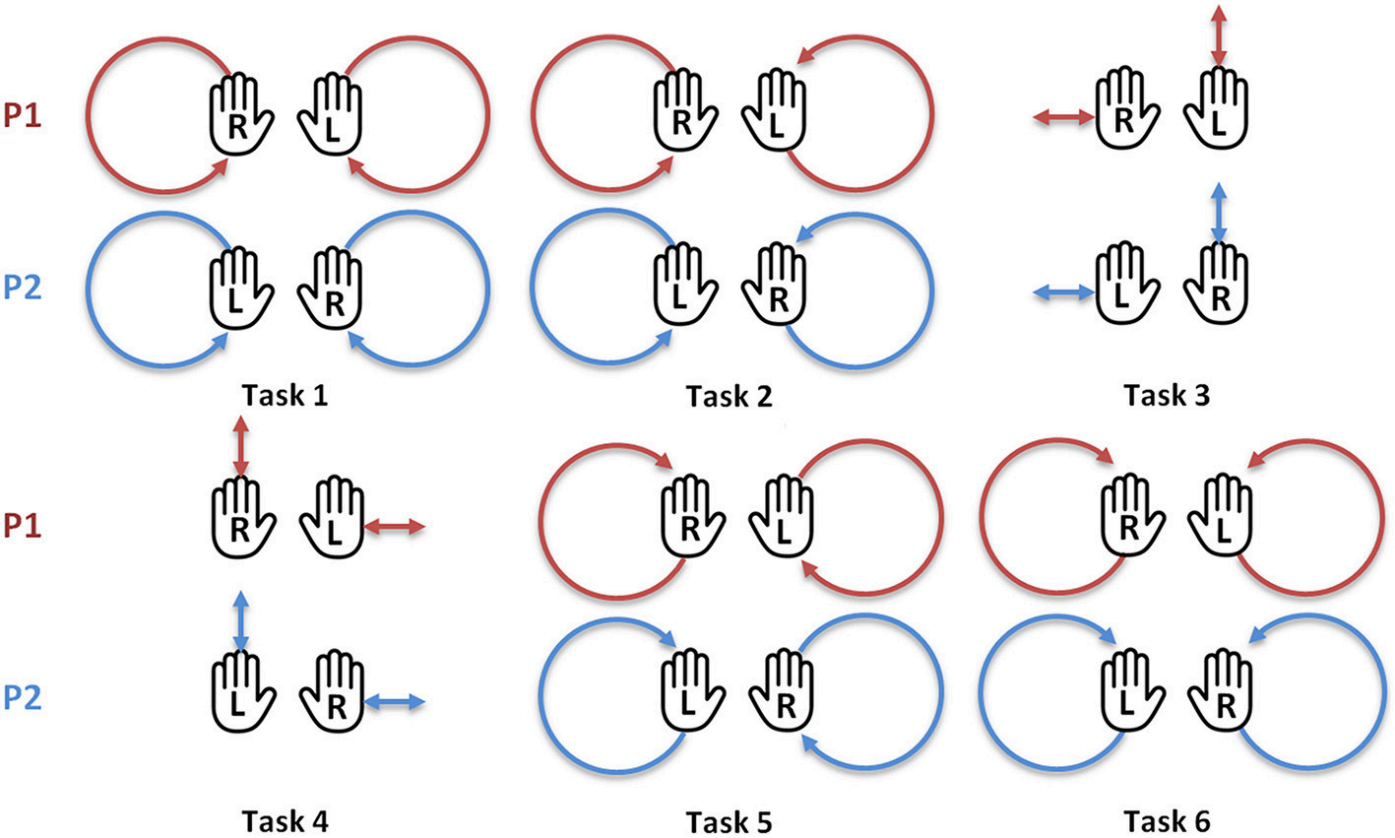
**The six motor tasks performed during the *blind* and *joint* motion sessions by the two participants**. Task 1: Participant 1 (P1) and participant 2 (P2) move the right hand in a clockwise circle and the left hand in a counterclockwise circle. Task 2: P1 moves both hands in clockwise circles, P2 moves both hands in counterclockwise circles. Task 3: P1 moves the right hand horizontally and the left hand vertically, P2 moves the right hand vertically and the left hand horizontally. Task 4: same as the task 3, but the participant's moves are reversed. Task 5: same as the task 2, but the participant's moves are reversed. Task 6: same as the task 1, but the participant's moves are reversed. R, right hand; L, left hand. Image reproduced from Llobera et al. (2016).

Participants were asked to stand opposite one another with a face-to-face distance of 80 cm to ensure that no physical contact could be made (see Figure 1). They performed two successive motion sessions (separated by a 10-min break) in two different conditions, *blind* and *joint*. Each session consisted of 6 motion tasks of 1 min, as detailed in Figure 2. The minimum frequency imposed to the subjects was around 0.3 Hz, but they could choose their own frequency to perform the task. To prevent an order effect, both the order in which the conditions (*blind, joint* vs. *joint, blind*) and the motor tasks (tasks 1–6 vs. tasks 6–1) were carried out was counterbalanced between dyads.

For the *blind* condition, the participants executed the tasks with a sleep mask placed on their eyes in order to perform the task without being distracted by the other participant's movements. The aim of this session was to gather baseline motion data on how they individually performed the tasks when no particular instruction was given.

For the *joint* condition, the participants were asked to execute the tasks in mirror. In order for the participants to not be influenced by the facial expression, appearance, or other factors, the overhead lights were turned off and the tasks were carried out in the dark. Visual cues were provided using two phosphorescent tapes (2 cm width; Wellys, reflective safety tape) attached around the wrists of each participant (see Figure 1). To prevent biases due to head motion or distraction, participants were instructed to look during the whole duration of the task at a phosphorescent tape (2 cm width and 4 cm in length) placed on the torso of the other participant. In addition, the lights were turned on again at the end of the task to prevent adaptation to dark.

While performing the task, participants were instructed to say aloud when they felt the sensation of being in synchrony with their partner, and when this sensation disappeared. For simplicity and clarity, the participants said their “first name” to report the appearance of the sensation and “NOT first name” to report its disappearance. The state of each of the two participants were recorded in log files for subsequent analysis using a custom software protocol (Llobera et al., 2016). The voice of the participants or any other sound was covered by the white noise. The task was validated only if both participants reported the sensation of synchrony during a common phase of at least 10 s; otherwise the task was repeated before continuing to the next (maximum of 4 attempts).

#### Video test session

Two months later, participants were recalled and watched 42 1 min point light display videos (Johansson, 1973; Fraiman et al., 2014; Llobera et al., 2016) of the motion experiment in a random order—the 6 videos of their own performances during the *joint* condition session and 36 videos of other dyads (6 dyads × 6 tasks) randomly chosen among their group (except for the musicians that watched the 6 videos of their own performances, 30 videos of the other dyads of their group and 6 videos of the control group). For the controls and couples, three dyads of their group were excluded from the viewing in order to limit the time required to complete the test and to avoid false response due to fatigue. Each video showed the dyad during the entire motor task represented by moving dots against a black background, which preserved kinematic information but without being able to recognize the participants. For each video, participants were asked to indicate: (Q1) the starting time (time code) when they thought that the two people were in synchrony; (Q2) if they were present in the video; and (Q3) to which extent they were confident in their response to question 2 (0: not sure at all, 10: completely sure). The test was performed on a 22 inch monitor with videos being played in full screen mode using a common video player (VLC, VideoLAN).

### Data processing

Motion data was post-processed in Vicon Blade software and exported for each task and each condition. Respiration data was downsampled from 256 to 120 Hz using MATLAB (The MathWorks Inc.,) to match the rate of the motion capture data. As in Llobera et al. (2016), the following measures were calculated:

A *footstep synchrony index* to extract spontaneous synchrony during the walking tasks. This was obtained by detecting footsteps from the four markers placed on each foot and then extracting the index from the phase difference between footsteps, as follows:

(1) $$\begin{array}{l} FSI=\;Abs\{Min[Abs\left(\varphi^{P1}-\varphi^{P2}\right),Abs\left((\varphi^{P1}+1)-\varphi^{P2}\right),\\ \;\;Abs\left((\varphi^{P1}-1)-\varphi^{P2}\right)]-0.25\}\text{*}4 \end{array}$$

Where φ^*Pi*^ is the phase for participant *i* between two footsteps, expressed between 0 and 1: 0 means that the timing of the footsteps was maximally different (equivalent to 90° of phase difference), 1 means that the feet of both participants were on the floor simultaneously, either the same foot or the contrary one. A figure illustrating this index can be found in Llobera et al. (2016).

A *respiration synchrony index* to extract respiration synchrony for each trial during the different tasks. It was extracted using the same mathematical expression than for footsteps.

The *hands distance* (*h_dist*) and the *hands speed difference* (*h_speed_diff*), adapted from Noy et al. (2011) to assess the behavioral synchronization of each couple at each time instant during the different tasks. The first movement descriptor intended to capture the difference in the hands position at a given moment, whereas the second intended to capture the difference in hand velocity at a given moment. They were obtained by projecting the hands positions (i.e., average position of the two markers placed on each hand) on the medial plane separating the two participants standing face-to-face, and were formally expressed as follows:

(2) $$h\_dist=\;\frac{\left||h_{left}^{P1}-h_{right}^{P2}||+||h_{right}^{P1}-h_{left}^{P2}|\right|}{2}$$

(3) $$h\_speed\_diff=\frac{1}{2}\frac{\left||s_{left}^{P1}-s_{right}^{P2}|\right|}{\left||s_{left}^{P1}+s_{right}^{P2}|\right|}+\frac{1}{2}\frac{\left||s_{right}^{P1}-s_{left}^{P2}|\right|}{\left||s_{right}^{P1}+s_{left}^{P2}|\right|}$$

Where ${\text{h}}_{\text{left}/\text{right}}^{\text{Pi}}$ is the 2D projection of the *left* or *right* hand position of participant *i* on the medial plane and ${\text{s}}_{\text{left}/\text{right}}^{\text{Pi}}$ is the speed vector of the *left* or *right* hand of participant *i* in the medial plane. The average value was then computed for all measures at increments of 1 s, the temporal precision of the subjective reports.

In addition, we determined different variables based on the subjective reports of synchrony stored in the log files. For each motion task and each participant in the dyad, we considered the first time (in seconds) they reported the sensation of synchrony during the task (*first trigger*). We also computed for each task the time between each participant's first trigger (*trigger difference*) to assess the time necessary for both participants to reach common synchrony, and the total duration the two participants had the feeling of synchrony (*total sync duration*).

### Statistical analysis

The statistical analyses were performed with the software package R (R Core Team, 2014), version 3.1.1 and SPSS software (IBM SPSS Statistics; version 21.0).

## Results

### Motion experiment

#### Questionnaires

##### Empathy

A two-way ANOVA (group and sex as factors) testing possible sex or group difference on the empathy score showed no group effect [*F*_(2, 46)_ = 0.66; *P* = 0.51], no sex effect [*F*(1, 46) = 1.41; *P* = 0.24] nor sex^*^group interaction [*F*_(2, 46)_ = 0.36; *P* = 0.69]. The degree of empathy had also no influence on the apparition of synchrony feeling as there was no correlation between the first *trigger* apparition and empathy scores (*r* = −0.16; *P* = 0.12). There was also no correlation between the total time spent in synchrony and empathy scores (*P* = 0.8).

##### PANAS questionnaires

To test possible difference between groups at the beginning of the experiment for the level of positive and negative activations, one-way ANOVAs on the scores of positive and negative activations were conducted. These analysis showed no significant difference between groups for positive [*F*_(2, 49)_ = 0.008; *P* = 0.99] and negative [*F*_(2, 49)_ = 0.42; *P* = 0.65] activations. To test possible difference between groups in emotional activation after performing joint and blind tasks, the same analyses were conducted on the scores of the PANAS questionnaires filled in after blind and joint tasks. This revealed that there was no difference between groups in positive [*F*_(2, 49)_ = 1.56; *P* = 0.21] and negative [*F*_(2, 49)_ = 3.09; *P* = 0.054] activations after the *blind* condition. There was also no difference between groups after the *joint* condition for positive [*F*_(2, 49)_ = 0.92; *P* = 0.4] and negative [*F*_(2, 49)_ = 0.29; *P* = 0.74] activations.

#### Implicit synchronization

At the beginning of the experiment, subjects were asked to walk without being informed that synchrony was measured. As in Llobera et al. (2016), we calculated from the *footstep synchrony index* a measure of implicit synchrony corresponding to the time spent walking in phase for more than 70% (0.7) or 90% (0.9) of the total walk duration (Table 1).

**Table 1:** **Mean (± SEM) percent of time spent in phase during the implicit synchrony walking task**.

	**Walk 1**		**Walk 2**	
	**Percent phase > 0.7**	**Percent phase > 0.9**	**Percent phase > 0.7**	**Percent phase > 0.9**
Controls	35% (±11%)	11% (±5%)	45% (±20%)	16% (±9%)
Couples	41% (±19%)	15% (±11%)	55% (±24%)	22% (±12%)
Musicians	65% (±27%)^*^	29% (±16%)^*^	49% (±20%)	19% (±11%)

*The percent of walk synchronization was significantly higher in musicians compared to controls during walk 1*.

* *P < 0.05*.

Table 1 suggest that musicians had higher synchronization in walk 1 and decreased their synchronization in walk 2. A 2-way repeated ANOVA (with groups as factor and walk1-walk2 as repeated measures) tested the effects of group and time course (from walk 1 to walk 2) on walk synchronization. For the 0.7 index of synchronization, there was no significant effect of the repeated measures [*F*_(1, 23)_ = 0.692, *P* = 0.41], no group effect [*F*_(2, 23)_ = 1.48, *P* = 0.247] but a significant interaction between groups and walks [*F*_(2, 23)_ = 6.64, *P* = 0.005] indicating that time course had different effect depending on the groups. Because of this interaction, separate repeated measures ANOVAs were conducted in each group. These analysis showed a significant effect of time course in controls [*F*_(1, 9)_ = 6.33, *P* = 0.033] reflecting an increase of synchronization between walk 1 and walk 2. In couples, there was no significant effect of the repeated measure [*F*_(1, 9)_ = 4.48, *P* = 0.063]. In musicians, there was a significant effect of the repeated measure [*F*_(1, 5)_ = 8.67, *P* = 0.32] reflecting a decrease in synchronization from walk 1 to walk 2 in this group. In the first walk, musicians had significantly higher implicit synchronization than the control group, as shown by a one-way ANOVA on the 0.7 index of synchrony showing a group effect [*F*_(2, 23)_ = 4.61; *P* = 0.021]. Because of this group effect, we conducted *post hoc* analysis (Bonferroni) that showed that musicians had higher walk synchronization than controls (*P* = 0.021), while there was no significant difference between controls and couples (*P* = 1) nor between couples and musicians (*P* = 0.076). Interestingly, this difference was no more present in the second walk [*F*_(2, 23)_ = 0.52; *P* = 0.59].

*For the 0.9 index of synchronization*, a two-way repeated measures ANOVA (with groups as factor and walk1-walk2 as repeated measures) tested the effects of group and time course on synchronization. This analysis showed no significant effect of the repeated measures [*F*_(1, 23)_ = 0.047, *P* = 0.83], no group effect [*F*_(2, 23)_ = 2.167, *P* = 0.137] but a significant interaction between groups and walks [*F*_(2, 23)_ = 8.76, *P* = 0.001]. Because of this interaction, separate repeated measures ANOVAs in each group were conducted, showing no significant effect of the time course in the control group [*F*_(1, 9)_ = 3.67, *P* = 0.087] and a in couples [*F*_(1, 9)_ = 4.87, *P* = 0.055] while there was a significant effect [*F*_(1, 5)_ = 6.82, *P* = 0.048] in musicians reflecting a decrease in synchronization from walk 1 to walk 2. Like for the 0.7 index, there was also a significant group difference for the higher index (0.9) of during walk 1 when analyzed separately as demonstrated by a one-way ANOVA (group as factor) showing a significant group effect [*F*_(2, 23)_ = 4.78; *P* = 0.018]. *Post hoc* analysis (Bonferroni) showed that musicians had higher walk synchronization than controls (*P* = 0.018) while there was no significant difference between controls and couples (*P* = 1) nor between couples and musicians (*P* = 0.076). There was finally no group difference in the second walk [*F*_(2, 23)_ = 0.7; *P* = 0.5].

#### Explicit synchronization

For the analysis, similar tasks (see material and methods) were grouped (tasks 1 and 6, tasks 2 and 5, tasks 3 and 4) and classified to take into account the level of difficulty in the analysis. Tasks 1 and 6 were thus considered as easy, tasks 2 and 5 as intermediate and tasks 3 and 4 as difficult. Indeed, as observed empirically during the motion experiments and according to the participant's feedbacks after the experiment, tasks 1 and 6 were considered as the easiest tasks, tasks 3 and 4 as tasks with intermediate difficulty and tasks 2 and 5 as the most difficult ones.

##### Joint vs. blind conditions

Comparing the *blind* vs. *joint* conditions revealed that the *hands distance* and the *hands speed difference* were considerably higher when participants performed the tasks during the *blind* condition compared to the *joint* condition in all groups (Table 2).

**Table 2:** **Mean (± SEM) hands distance and hands speed difference during *blind* and *joint* conditions**.

	**Hands distance**		**Hands speed difference**	
	**Blind**	**Joint**	**Blind**	**Joint**
Controls	279.14 (±10.8)^*^	104.47 (±4.8)	1.77 (±0.15)^*^	0.28 (±0.02)
Couples	287.5 (±12.4)^*^	103.6 (±5.1)	1.88 (±0.17)^*^	0.3 (±0.02)
Musicians	319.1 (±17.2)^*^	101.4 (±6.8)	2.22 (±0.26)^*^	0.26 (±0.02)

*Distance and speed differences were higher in all groups during the blind condition compared to the joint condition*.

* *P < 0.05*.

For the *hands distance*, a three-way ANOVA (with groups, tasks and *joint/blind* as factors) was conducted to control that *blind* conditions were actually giving rise to less synchronization than *joint* tasks. This analysis confirmed that there was a strong effect of *joint* vs. *blind* conditions [*F*_(1, 156)_ = 516.42; *P* < 0.0001], there was also a significant effect of tasks [*F*_(2, 156)_ = 7.21; *P* = 0.001] but no significant interaction between groups and tasks [*F*_(4, 156)_ = 0.731; *P* = 0.572] nor between tasks and blind/joint conditions [*F*_(2, 156)_ = 2.94, *P* = 0.056]. For the *hands speed difference*, there was no difference between groups as revealed by a three-way ANOVA (with groups, tasks and *joint/blind* as factors) showing a strong effect of *joint* vs. *blind* conditions [*F*_(1, 156)_ = 238.68, *P* < 0.0001], a significant effect of tasks [*F*_(2, 156)_ = 4.32, *P* = 0.015] but no significant interaction between groups and tasks [*F*_(4, 156)_ = 1.23, *P* = 0.3] nor between tasks and *blind/joint* conditions [*F*_(2, 156)_ = 0.92, *P* = 0.39].

##### First trigger apparition and time difference between triggers apparition in each dyad

The feeling of synchrony appeared earlier in musicians as shown by Table 3.

**Table 3:** **Mean (± SEM) time in seconds of the *first trigger* of synchrony apparition, the difference in triggers apparition and the total duration of synchrony feeling**.

	**First trigger apparition**	**Triggers difference**	**Total sync duration**
Controls	21.09 (±1.8)	7.72 (±1.2)	36.32 (±1.66)
Couples	17.06 (±1.03)	6.25 (±1.01)	37.37 (±1.47)
Musicians	14.54 (±1.12)^*^	4.97 (±0.98)	39.82 (±3.2)

*The feeling of synchrony appeared significantly earlier in musicians compared to controls*.

* *P < 0.05*.

For the first *trigger apparition*, a two-way ANOVA (with group as factor and tasks as repeated measure) testing possible group difference in the apparition of the synchrony feeling in the different joint tasks showed no effect of tasks [*F*_(2, 98)_ = 2.87, *P* = 0.067] suggesting that the difficulty of tasks had no effect on the apparition of synchrony feeling. There was a significant group effect [*F*_(2, 49)_ = 4.8, *P* = 0.012] and no interaction between group and tasks [*F*_(4, 98)_ = 0.826, *P* = 0.512]. Because of the significant group effect, *post hoc* analyses (Bonferroni) were conducted showing that only the difference between controls and musicians was significant (*P* = 0.014) whereas there was no significant difference between musicians and couples (*P* = 0.78) nor between couples and controls (*P* = 0.12). The time difference between the sensation onsets of each member of the dyad was also shorter in the musicians group indicating that they felt the sensation of synchrony more at the same time.

Finally, for the total duration felt in synchrony, a two-way ANOVA (with group as factor and tasks as repeated measure) was conducted to test possible group difference in the duration of the synchrony feeling in the different joint tasks. This analysis showed a significant effect of tasks difficulty [*F*_(2, 98)_ = 11.78, *P* < 0.0001], no group effect [*F*_(2, 49)_ = 0.707, *P* = 0.498] and no interaction between tasks and group [*F*_(4, 98)_ = 0.763, *P* = 0.552]. Thus, there was no group effects on the total time that subjects reported to have spent in synchrony but tasks difficulty had an effect on the total time spent in synchrony (mean ± SEM; tasks 1 and 6 as easy tasks (39.57 ± 1.27), tasks 2 and 5 as intermediate tasks (34.5 ± 1.23) and tasks 3 and 4 as difficult tasks (38.5 ± 1.23). Thus, the intermediate tasks were associated with lower time spent in synchrony.

##### Relations between the quality of interactions in romantic couples and the feeling of synchrony

**Figure 3:**
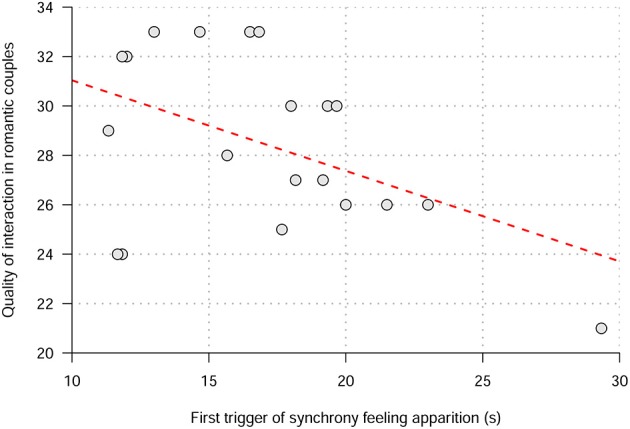
**Correlation between the quality of dyadic interactions in romantic couples (measured by DAS questionnaires) and the first trigger apparition of synchrony**.

To assess the quality of the relationship in romantic couples, we used the abbreviated form of the Dyadic Adjustment Scale (DAS) assessing two different dimensions: the degree of agreement and the quality of the dyadic interactions. Correlation analyses were conducted to explore possible relations between dyadic adjustment, the feeling of synchrony and the behavioral synchronization. There was a negative unilateral Pearson correlation between the quality of dyadic interactions and the first *trigger* apparition (*r* = −0.46; *P* = 0.019) and difference between triggers apparition (*r* = −0.4; *P* = 0.039). Thus, the more couples had a good quality of dyadic interactions the less time it took them to feel in synchrony and the less was the difference of time between their feeling of synchrony (Figure 3). To ensure that the observation on the right of the figure was not too influential, we calculated the Cook distance (D = 0.533) confirming that this observation was not an outlier (as the Cook distance was under the threshold of 1 considered to reflect too influential cases). However, removing this observation from the correlational analysis will result in a non-significant correlation (*p* > 0.05) suggesting that these correlation results should be taken with caution.

There was no correlation between the first *trigger* and the degree of accord (*P* = 0.3). Interestingly, there was a nearly significant negative correlation between the quality of interactions and hand speed difference (*P* = 0.05), suggesting that the more couples had a good quality of interaction, the better was the behavioral synchrony of hand speed.

Finally, there was no correlation between the degree of accord and *hands speed difference* (*P* = 0.9). For the attachment styles, there was no significant unilateral Pearson correlation between the first *trigger* apparition and all attachment styles (F score (*r* = −0.036; *P* = 0.44), D score (*r* = −0.07; *P* = 0.37), S score (*r* = −0.006; *P* = 0.49) and P score (*r* = 0.18; *P* = 0.22).

#### Objective measures of synchronization: hands distance and speed difference

**Figure 4:**
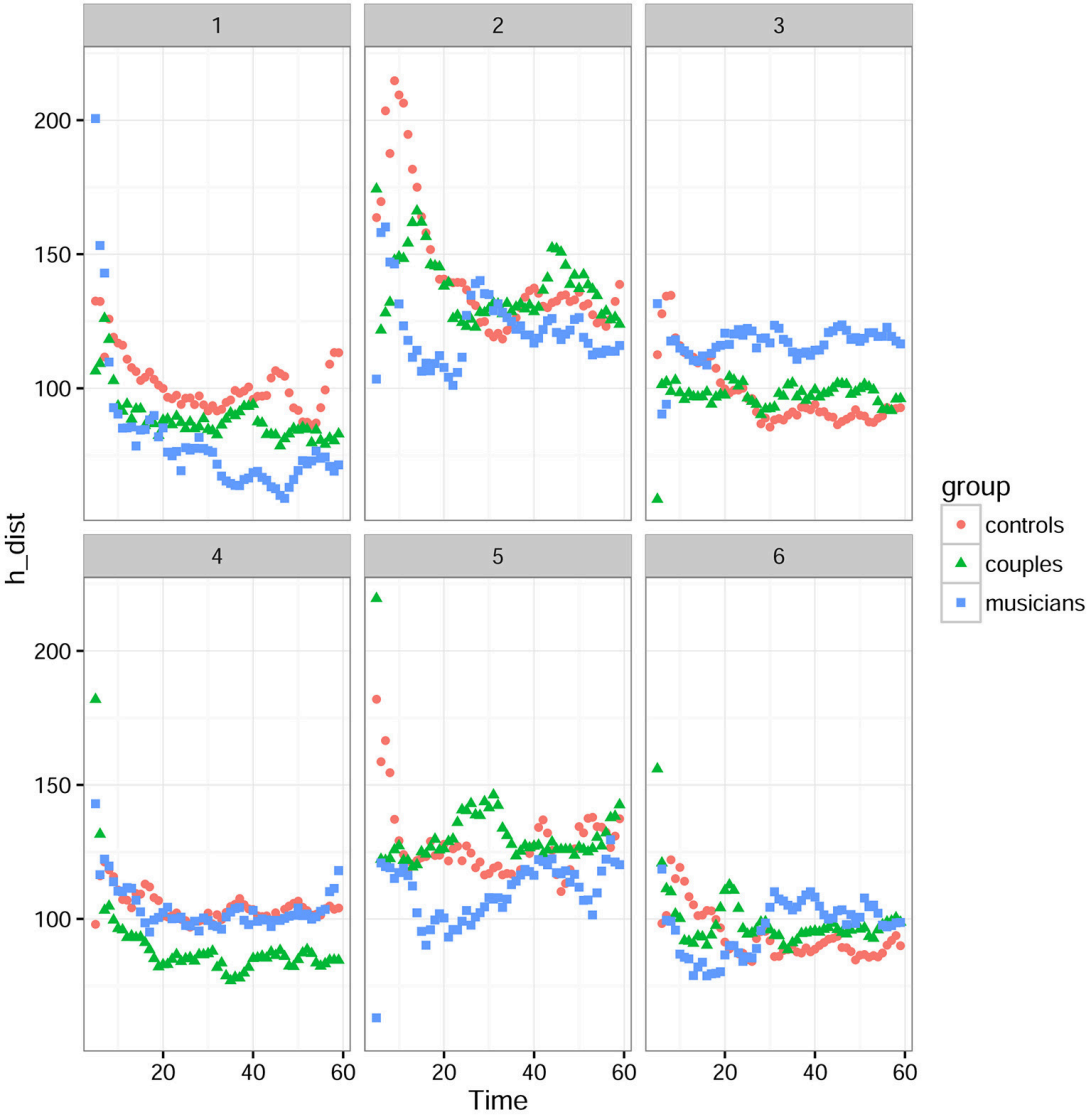
**Mean *hand distances* for tasks 1 to 6 in each group**. 0–20 s is the starting phase, 20–40 s the stabilization phase, and 40–60 s the ending phase.

Figure 4 shows that there was a strong interaction between the difficulty of the tasks and the groups. For this analysis, the tasks were divided into three periods: (1) 0–20 s corresponding to the “starting phase” where participants start the task and look for synchronization, (2) 20–40 s corresponding to a “stabilization phase” where participants are synchronized and feel the synchrony sensation, and (3) 40–60 s corresponding to the “ending phase.” Statistical analysis was conducted in the time window of the second period, since it was the most significant.

For the *hands distance*, a linear mixed-effects model with group and task difficulty as factors was used to test if the behavioral synchronization differed between groups depending on the difficulty of the tasks. This analysis revealed that there was a significant effect of difficulty [*F*_(2, 8295.5)_ = 787.32; *P* < 0.0001] no significant group effect [F_(2, 26)_ = 0.27; *P* = 0.76] and a significant interaction between groups and difficulty [*F*^(4, 8295.5)^ = 59.05; *P* < 0.0001]. Because of the significant interaction between groups and difficulty, separate analysis in tasks grouped by difficulty were conducted. However, this separate analysis with a linear mixed-effects model showed no significant difference between groups for the easiest tasks (tasks 1 and 6) [*F*_(2, 26)_ = 0.39, *P* = 0.679], the tasks with intermediate difficulty (tasks 2 and 5) [*F*_(2, 26)_ = 0.694; *P* = 0.509] and the most difficult ones (tasks 3 and 4) [*F*_(2, 26)_ = 1.119; = 0.342).

**Figure 5:**
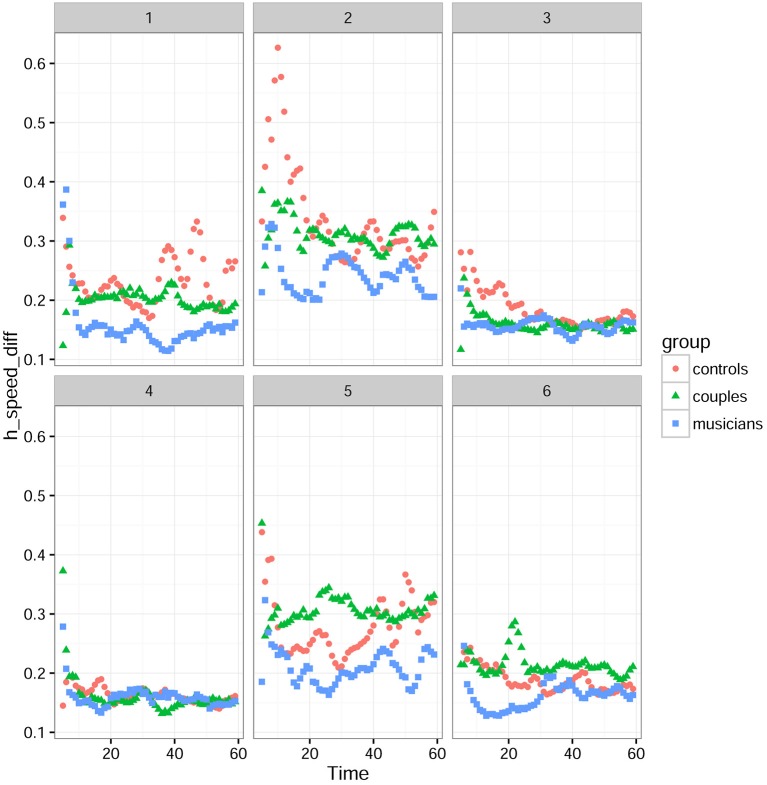
**Mean *hand speed differences* for tasks 1 to 6 in each group**. 0–20 s is the starting phase, 20–40 s the stabilization phase, and 40–60 s the ending phase.

Figure 5 shows however that musicians had lower speed errors compared to controls and couples. For the *speed errors*, a linear mixed-effects model with group and task difficulty as factors revealed that there was a significant effect of difficulty [F_(2, 8295)_ = 1128), *P* < 0.001), no significant group effect [F_(2, 26)_ = 3.01, *P* = 0.0665] and a significant interaction between groups and difficulty [*F*_(4, 8295.5)_ = 59.05; *P* < 0.0001]. Because of the significant interaction between groups and difficulty, separate analysis in tasks grouped by difficulty were conducted. This separate analysis with a linear mixed-effects model showed no significant difference between groups for the easiest tasks (tasks 1 and 6) [*F*_(2, 26)_ = 2.58, *P* = 0.095], a significant difference for the tasks with intermediate difficulty (tasks 2 and 5) [*F*_(2, 26)_ = 4.37; *P* = 0.023] but not for the most difficult ones (tasks 3 and 4) [*F*_(2, 26)_ = 0.51; = 0.607).

##### Respiration synchronization

For the respiration, comparing the *blind* vs. *joint* conditions showed no difference in all groups (*joint* condition, controls: 0.5 ± 0.019; couples: 0.51 ± 0.02; musicians: 0.49 ± 0 and *blind* condition, controls: 0.5 ± 0.022; couples: 0.049 ± 0.02; musicians 0.51 ± 0.01) for mean phase differences. This was confirmed by a repeated measure ANOVA with *joint*/*blind* conditions as repeated measure that showed no difference between *joint* and *blind* conditions [*F*_(1, 23)_ = 0.2; *P* = 0.88], no group effect [*F*_(2, 23)_ = 0.02; *P* = 0.97]. As for the analysis of footsteps synchronization, we calculated from the *respiration synchrony index* a measure of synchrony corresponding to the time spent breathing in phase for more than 70% (0.7) of the total task duration. Again, there was no difference between the *joint* and *blind* conditions nor between groups (*joint* condition, controls: 0.3 ± 0.02; couples: 0.32 ± 0.03; musicians: 0.29 ± 0.02 and *blind* condition, controls: 0.3 ± 0.03, couples: 0.28 ± 0.03; musicians 0.31 ± 0.01) for the percent of phase synchrony over 0.7. This was confirmed by a repeated measure ANOVA with *joint*/*blind* conditions as repeated measure that showed no difference between *joint* and *blind* conditions [*F*_(1, 23)_ = 0.17; *P* = 0.67], no group effect [*F*_(2, 23)_ = 0.03; *P* = 0.96]. Since respiration did not show any sensitivity to the fact that the task was done jointly or separately and was not different across groups, we excluded respiration data from further analysis.

### Video test session

For each video, participants were asked to indicate: (Q1) the starting time (time code) when they thought that the two people were in synchrony; (Q2) if they were present in the video; and (Q3) to which extent they were confident in their response to question 2 (0: not sure at all, 10: completely sure). We built a first generalized linear mixed model (McCullagh and Nelder, 1989; Dobson and Barnett, 2008; Faraway, 2016) to evaluate how a viewer is able to determine the instant at which both members of a couple are in synchrony (question Q1 of the test). Similar to Llobera et al. (2016), to relate the video test reports with the subjective reports on synchrony feeling, we built a predictor variable named *trigger*. The reason to introduce such predictor variable was because the subjective reports of synchrony sometimes had short onsets. In addition, one of the conditions to validate a trial was that the feeling of synchrony lasted more than 10 s. Therefore, since both participants had given different responses to the moment where they perceived to be in synchrony, the predictor variable named trigger was calculated from the mean of first trigger (first appearance of the synchrony feeling) and common trigger (feeling of synchrony for more than 10 s). Thus, for each participant in the couple, we considered the first time they reported the sensation during the task, and the time they reported the sensation that validated the trial (i.e., when this sensation occurred during a common phase of at least 10 s, which could be different from the first time the sensation occurred). Additional predictor variables considered were: the task (*task*), the fact that the respondent was present or not in the video (*presence*), the gender of the respondent (*gender*), the level of empathy of the respondent (*empathy*) and the Panas questionnaires (*Panas_pos*; *Panas_neg*) of the respondent.

Using this linear mixed-effects model with *trigger, task, gender, empathy, Panas_pos, Panas_neg*, the *presence* on the video and *group* as explanatory variables, we showed that concerning the ability to identify the instant when the dyad was in synchrony (Q1), only the *trigger* variable corresponding to the moment when both partners were synchronized was significant [*F*_(1, 1825)_ = 39.61; *P* < 0.001] among all potentially explanatory variables of the participant's response to the question Q1. In other words, the only variable that was significant was the *trigger* variable corresponding to the moment where the apparition of synchrony was reported. Thus, participants looking at the videos with a third person perspective were able to identify the moment reported (*trigger*) by participants from a first person perspective. There was no group effect [*F*_(2, 48)_ = 1.912; *P* = 0.159] nor sex effect [*F*_(1, 48)_ = 0.1; *P* = 0.753]. Being present in the video [*F*_(1, 1822)_ = 1.46; *P* = 0.227] did not influence this capacity.

Participants were able to recognize if they were present or not in the video (Q2). Using a logistic mixed-effects model with the *presence, tasks, gender, empathy, Panas_pos, Panas_neg* and *group* as explanatory variables, only the fact of being present in the video was significant (χ^2^(1) = 13.96, *P* < 0.001) whereas there was a non-significant tendency of a group effect (χ^2^(2) = 5.72, *P* = 0.057). Simultaneous tests for general linear hypotheses in *post hoc* analyses showed that musicians were better to recognize themselves compared to controls (*z* = 2.45, *P* = 0.038) but not compared to couples (*z* = 1.24, *P* = 0.429) and there was no difference between controls and couples (*z* = 1.42, *P* = 0.332).

Finally, responses to Q3 seemed to appropriately reflect the level of confidence that participants had in their responses. Indeed, their level of confidence in answering Q3 was significantly higher when they answered correctly to Q2 than when they did not (χ^2^(1) = 39.78, *p* < 0.001) without group effect (χ^2^(2) = 3.62, *p* = 0.164).

## Discussion

Our experiment included both implicit (spontaneous synchronization during walking) and explicit tasks (mirror motor tasks). Motion capture data allowed to link objective measures of synchronization with the subjective feeling of synchrony in each group. Finally, the video test session allowed to investigate possible group effect on the capacity to recognize synchrony from a third person perspective. Our hypothesis was that body synchronization and the feeling of synchrony should appear earlier in musicians and people in a romantic relationship. Contrary to our hypothesis, we did not find clear differences between people in a romantic relationship and control subjects. However, we observed significant difference between musicians and control subjects for implicit and explicit synchrony (both for the feeling of synchrony and behavioral synchronization assessed by motion capture), as well as third perspective synchrony observation.

For the *implicit synchrony*, our results show that dyads of musicians that had never met before synchronized their behavior better and faster than control subjects, even if not explicitly asked to do so. During the first walk, musicians had higher synchrony index, both for the 70 and 90% measures. This suggests that musicians, even when not explicitly asked to, had a more rapid and effective tendency to spontaneously synchronize themselves, compared to non-musician control subjects. However, this effect did not last longer as there was no difference during the second walk. This was mainly due to an interaction between the two walks and groups. Indeed, while musicians had higher synchrony during the first walk, their level of synchrony decreased in second walk, while controls had exactly the inverted profile (they had lower synchrony in the first walk and increased their level of synchrony during the second walk).

For the *explicit synchrony* (mirror game), control conditions in which subjects performed the tasks without seeing each other (*blind* condition) confirmed that subjects in all groups had higher hands distance and speed difference compared to doing the tasks together (*joint* condition). Interestingly, in the *joint* condition, musicians had higher behavioral synchronization than controls, particularly in the speed errors as there was no group effect in the hands distance. Thus, speed differences seems to be a better measure of possible intergroup differences in synchronization than hands distance. There was also a significant interaction with the tasks. Indeed, the better performance of musicians was only visible in the tasks with intermediate difficulty (tasks 2 and 5). These tasks were also the tasks associated with the less time spent in synchrony in all groups. Thus, in coherence with our previous results (Llobera et al., 2016), the tasks difficulty seems to play also a role in intergroup differences for behavioral synchronization and the feeling of synchrony. One can hypothesize that we observed the difference between musicians and controls in intermediate difficulty tasks because easy tasks might have been too easy and all groups performed them well (floor effect) and the more difficult tasks might have induced a ceiling effect being too difficult for all groups, thus preventing from seeing differences. In this way, intermediate difficulty tasks might be the best tasks to use to study possible intergroup differences.

The *feeling of synchrony* appeared earlier in musicians than in control subjects. The difference of time in the synchrony feeling onset between the two members of the dyad was also reduced compared to controls (i.e., they started feeling synchrony closer in time). In romantic couples, there was a negative correlation between the quality of dyadic interactions in the couple and both the first trigger apparition and the difference between triggers apparition. In other words, the more the couples had a good quality of interactions, the less time it took them to have the feeling of synchrony and the smaller was the difference of time between their feelings of synchrony. It suggests that objective measures on a joint behavior task can predict subjective interrelation factors, such as the ones reflected by the DAS questionnaire. This, though, has to be taken with caution, since it is known that correlation does not imply causation and other underlying factors could be at play. Moreover, since we had a reduced number of subjects, this correlation should be taken with caution as it could have been influenced by extreme value (removing one couple resulted in non-significant results). Further studies are thus needed to clarify whether this objective measure can be used. Taken together, our results show that the group of people in a romantic relationship taken as a whole were not significantly different than control subjects for the apparition of the synchrony feeling nor for the bodily synchronization during implicit and explicit tasks. Thus, being in a romantic relationship for 1 year might not be sufficient to increase synchrony.

The difference we observed between musicians and control groups for the onset of the feeling of synchrony does not seem to rely on empathy differences. Indeed, there was no significant difference between groups nor sex differences for the level of empathy. Moreover, there was no correlation between the onset time and empathy scores. Synchrony seems also not to be mediated by positive emotions as there was no difference in the positive activations before and after the synchronization task. This is in line with previous experiments showing that effects of synchrony on prosocial and cooperative behaviors are not mediated by positive emotions (Wiltermuth and Heath, 2009). Moreover, we did not find any synchronization in respiration in all groups. This is in accord with our previous results in control subjects only (Llobera et al., 2016) and it suggests that respiration might not be a useful predictor of the subjective feeling of synchrony, at least for such kind of imitation tasks.

Concerning the *recognition of synchrony* from a third-person perspective, all participants were able to identify the moment of synchrony as reported by the people doing the motor tasks. Moreover, being present in the video did not influence this capacity. This is in line with our previous results (Llobera et al., 2016). In addition, in the present experiment we could not find any group difference in the ability to recognize the onset of synchrony. This suggests that although musicians felt the synchrony earlier during the tasks and had better motor synchronization especially for speed adjustment, they were not better than control subjects to detect synchrony from a third person perspective. In contrast, musicians were better than controls to recognize if they were effectively present in the video. The better performance of musicians could rely on the fact that they do joint performance more often. This could also explain why our control subjects were not very good in determining if they were present in the video despite the fact that previous studies have found that people are able to recognize themselves in this type of point light displays (Sevdalis and Keller, 2009). Indeed, these studies mainly used tasks involving individual actions whereas our tasks involved joint action recognition. Thus, recognizing our presence in joint action videos might involve different mechanisms than the ones for simple individual actions. Finally, there was a significant relationship between the level of confidence in this response and the actual performance in all groups suggesting that subjects had a good insight about their actual abilities to recognize their presence on the videos.

Our results showed that musicians had better behavioral synchronization and reported the feeling of synchrony earlier than control subjects. Musicians might be more demanding to explicitly report to be in synchrony and more sensitive to small synchrony default (Steinbeis et al., 2006). Previous studies have shown that the musician's ability to synchronize is strongly supported by the representation and the anticipation of the other musician's behavior and anticipatory auditory image. For instance, pianists are better to synchronize with their own music (when replayed) compared to the same music played by someone else suggesting that anticipation and representation of the movements is a crucial key of synchronization (see Keller et al., 2014 for a review). It is also possible that the feeling of synchrony is affected by music. Indeed, Demos et al. (2012) showed that people in rocking chairs reported higher synchrony when they tried to synchronize with music than when they tried to synchronize without music, despite the fact that the level of synchrony was actually lower. It also seems consistent with what Reddish et al., 2013) consider one of the main functions of music, singing and dancing: social bonding. Overall, it suggests that behavioral synchrony *per se* might not be the only source of the feeling of synchrony and other factors such as music might also play a role, irrespectively of people's musical training.

Our results also suggest that objective measures based on motion capture data can be used to predict the appearance of the feeling of synchrony and suggest that bodily factors play an important role in the feeling of synchrony. Bodily factors have previously been proposed to play an important role in other subjective feeling and cognitive functions including decisions making, memory, emotions and drive (Damasio and Carvalho, 2013; Arminjon et al., 2015; Magistretti and Ansermet, 2016) and body information should be more taken into account in neuroscience studies (de Gelder, 2009). However, our study also involves certain limitations. Indeed, despite we used several measures to assess factors affecting the subjective feeling of synchrony, our number of subjects was limited. Moreover, we selected professional musicians that all had an experience of playing in a group music for several years. Thus, it is difficult to disentangle if the increase in synchrony was due to musical training only or to the experience of playing with other musicians. One way to disentangle this could be in further studies to compare musicians playing in group with for instance people working in synchronization with others during their work or basketball players that have to synchronize with others in the absence of music. There are to our knowledge no study that did disentangle this question of music and synchronization (indeed, the better synchrony observed in dancers might be at least partially due to music). Finally, we had a strong interaction with the type of tasks used to induce synchrony. Indeed, musicians were better in synchronization and felt synchrony earlier only in intermediate difficulty tasks that were the tasks that provoked less total synchrony feeling. Thus, the type of tasks used to induce synchrony with behavioral synchronization seem to play an important role. Overall, since there have been very few studies on this subject, further research is needed to more precisely understand the role of interpersonal or intrapersonal factors on the subjective feeling of synchrony and its correlates.

## Author contributions

Conceived and designed the experiments: JL, CC, DP, FA, and PM; Performed the experiments: CC and SC; Analyzed the data: DP, JA, CC, and SC; Contributed reagents/materials/analysis tools: DP, JA, CC, SC, and JL; Wrote the paper: DP and CC.

## Funding

This research project has been entirely funded by the Agalma Foundation. The funder had no role in study design, data collection and analysis, decision to publish, or preparation of the manuscript.

### Conflict of interest statement

The authors declare that the research was conducted in the absence of any commercial or financial relationships that could be construed as a potential conflict of interest.

## References

[B1] Antoine P. Christophe V. Nandrino J. (2008). Échelle d'ajustement dyadique : intérêts cliniques d'une révision et validation d'une version abrégée. L'Encéphale 34, 38–46. 10.1016/j.encep.2006.12.005 18514149

[B2] Arminjon M. Preissmann D. Chmetz F. Duraku A. Ansermet F. Magistretti P. J. (2015). Embodied memory: unconscious smiling modulates emotional evaluation of episodic memories. Front. Psychol. 6:650. 10.3389/fpsyg.2015.00650 26074833 PMC4443770

[B3] Carpenter M. Uebel J. Tomasello M. (2013). Being mimicked increases prosocial behavior in 18-month-old infants. Child Dev. 84, 1511–1518. 10.1111/cdev.12083 23488734

[B4] Chartrand T. L. Bargh J. A. (1999). The chameleon effect: the perception-behavior link and social interaction. J. Pers. Soc. Psychol. 76, 893–910. 10.1037/0022-3514.76.6.893 10402679

[B5] Clayton M. (2009). The social and personal functions of music in cross-cultural perspective, in The Oxford Handbook of Music Psychology, eds Hallam S. Cross I. Thaut M. (Oxford: Oxford University Press), 3–44.

[B6] Damasio A. Carvalho G. B. (2013). The nature of feelings: evolutionary and neurobiological origins. Nat. Rev. Neurosci. 14, 143–152. 10.1038/nrn3403 23329161

[B7] Davis M. H. (1980). A multidimensional approach to individual differences in empathy. JSAS Catalog Select. Doc. Psychol. 10, 85.

[B8] de Gelder B. (2009). Why bodies? Twelve reasons for including bodily expressions in affective neuroscience. Philos. Trans. R. Soc. B. Biol. Sci. 364, 3475–3484. 10.1098/rstb.2009.0190 19884142 PMC2781896

[B9] Demos A. P. Chaffin R. Begosh K. T. Daniels J. R. Marsh K. L. (2012). Rocking to the beat: effects of music and partner's movements on spontaneous interpersonal coordination. J. Exp. Psychol. Gen. 141, 49–53. 10.1037/a0023843 21668129

[B10] Dobson A. J. Barnett A. (2008). An Introduction to Generalized Linear Models. Boca Raton, FL: CRC press.

[B11] Faraway J. J. (2016). Extending the Linear Model with R: Generalized Linear, Mixed Effects and Nonparametric Regression Models. Vol. 124 Boca Raton, FL: CRC Press.

[B12] Fraiman D. Saunier G. Martins E. F. Vargas C. D. (2014). Biological motion coding in the brain: analysis of visually driven EEG functional networks. PLoS ONE 9:e84612. 10.1371/journal.pone.0084612 24454734 PMC3891803

[B13] Gallese V. (2003). The roots of empathy: the shared manifold hypothesis and the neural basis of intersubjectivity. Psychopathology 36, 171–180. 10.1159/000072786 14504450

[B14] Gaudreau P. Sanchez X. Blondin J. (2006). Positive and negative affective states in a performance-related setting. Eur. J. Psychol. Assess. 22, 240–249. 10.1027/1015-5759.22.4.240

[B15] Griffin D. W. Bartholomew K. (1994). Models of the self and other: fundamental dimensions underlying measures of adult attachment. J. Pers. Soc. Psychol. 67, 430–445. 10.1037/0022-3514.67.3.430

[B16] Hagen E. H. Bryant G. A. (2003). Music and dance as a coalition signaling system. Hum. Nat. 14, 21–51. 10.1007/s12110-003-1015-z 26189987

[B17] Hart Y. Noy L. Feniger-Schaal R. Mayo A. E. Alon U. (2014). Individuality and togetherness in joint improvised motion. PLoS ONE 9:e87213. 10.1371/journal.pone.0087213 24533054 PMC3922750

[B18] Hove M. J. Risen J. L. (2009). It's all in the timing: interpersonal synchrony increases affiliation. Soc. Cogn. 27, 949–960. 10.1521/soco.2009.27.6.949

[B19] Iacoboni M. Molnar-Szakacs I. Gallese V. Buccino G. Mazziotta J. C. Rizzolatti G. (2005). Grasping the intentions of others with one's own mirror neuron system. PLoS Biol. 3:e79. 10.1371/journal.pbio.0030079 15736981 PMC1044835

[B20] Johansson G. (1973). Visual perception of biological motion and a model for its analysis. Percept. Psychophys. 14, 201–211. 10.3758/BF03212378

[B21] Karremans J. C. Verwijmeren T. (2008). Mimicking attractive opposite-sex others: the role of romantic relationship status. Pers. Soc. Psychol. Bull. 34, 939–950. 10.1177/0146167208316693 18453390

[B22] Keller P. E. Novembre G. Hove M. J. (2014). Rhythm in joint action: psychological and neurophysiological mechanisms for real-time interpersonal coordination. Philos. Trans. R. Soc. B. Biol. Sci. 369, 20130394–20130394. 10.1098/rstb.2013.0394 25385772 PMC4240961

[B23] Kirschner S. Tomasello M. (2010). Joint music making promotes prosocial behavior in 4-year-old children. Evol. Hum. Behav. 31, 354–364. 10.1016/j.evolhumbehav.2010.04.004

[B24] Knoblich G. Butterfill S. Sebanz N. (2011). Psychological research on joint action: theory and data. The Psychology of Learning and Motivation, ed Ross B. (Burlington, MA: Academic Press), 59–101.

[B25] Llobera J. Charbonnier C. Chagué S. Preissmann D. Antonietti J. P. Ansermet F.. (2016). The Subjective sensation of synchrony: an experimental study. PLoS ONE 11:e0147008. 10.1371/journal.pone.0147008 26870943 PMC4752214

[B26] Lumsden J. Miles L. K. Richardson M. J. Smith C. A. Macrae C. N. (2012). Who syncs? Social motives and interpersonal coordination. J. Exp. Soc. Psychol. 48, 746–751. 10.1016/j.jesp.2011.12.007

[B27] Magistretti P. J. Ansermet F. (2016). The island of drive: representations, somatic states and the origin of drive, in A Neuro-Psychoanalytical Dialogue for Bridging Freud and the Neurosciences, eds Weigel S. Scharbert G. (Cham: Springer International Publishing), 137–147.

[B28] McCullagh P. Nelder J. A. (1989). Generalized Linear Models. London: Chapman and Hall.

[B29] Noy L. Dekel E. Alon U. (2011). The mirror game as a paradigm for studying the dynamics of two people improvising motion together. Proc. Natl. Acad. Sci.U.S.A. 108, 20947–20952. 10.1073/pnas.1108155108 22160696 PMC3248496

[B30] Noy L. Levit-Binun N. Golland Y. (2015). Being in the zone: Physiological markers of togetherness in joint improvisation. Front. Hum. Neurosci. 9:187. 10.3389/fnhum.2015.00187 25999832 PMC4419713

[B31] Ramseyer F. Tschacher W. (2011). Nonverbal synchrony in psychotherapy: coordinated body movement reflects relationship quality and outcome. J. Consult. Clin. Psychol. 79, 284–295. 10.1037/a0023419 21639608

[B32] Ramseyer F. Tschacher W. (2014). Nonverbal synchrony of head- and body-movement in psychotherapy: different signals have different associations with outcome. Front. Psychol. 5:979. 10.3389/fpsyg.2014.00979 25249994 PMC4155778

[B33] R Core Team (2014). R: A Language and Environment for Statistical Computing. R Foundation for Statistical Computing,

[B34] Reddish P. Fischer R. Bulbulia J. (2013). Let's dance together: synchrony, shared intentionality and cooperation. PLoS ONE 8:e71182. 10.1371/journal.pone.0071182 23951106 PMC3737148

[B35] Repp B. H. (2005). Sensorimotor synchronization: a review of the tapping literature. Psychon. Bull. Rev. 12, 969–992. 10.3758/BF03206433 16615317

[B36] Sebanz N. Bekkering H. Knoblich G. (2006). Joint action: bodies and minds moving together. Trends Cogn. Sci. 10, 70–76. 10.1016/j.tics.2005.12.009 16406326

[B37] Sevdalis V. Keller P. E. (2009). Self-recognition in the perception of actions performed in synchrony with music. Ann. N. Y. Acad. Sci. 1169, 499–502. 10.1111/j.1749-6632.2009.04773.x 19673830

[B38] Spanier G. B. (1976). Measuring dyadic adjustment: new scales for assessing the quality of marriage and similar dyads. J. Marriage Fam. 38, 15 10.2307/350547

[B39] Steinbeis N. Koelsch S. Sloboda J. A. (2006). The role of harmonic expectancy violations in musical emotions: evidence from subjective, physiological, and neural responses. J. Cogn. Neurosci. 18, 1380–1393. 10.1162/jocn.2006.18.8.1380 16859422

[B40] Stel M. van Baaren R. B. Blascovich J. van Dijk E. McCall C. Pollmann M. M.. (2010). Effects of a priori liking on the elicitation of mimicry. Exp. Psychol. 57, 412–418. 10.1027/1618-3169/a000050 20178935

[B41] Trainor L. J. Cirelli L. (2015). Rhythm and interpersonal synchrony in early social development. Ann. N. Y. Acad. Sci. 1337, 45–52. 10.1111/nyas.12649 25773616

[B42] van Baaren R. B. Holland R. W. Kawakami K. Knippenberg A. V. (2004). Mimicry and prosocial behavior. Psychol. Sci. 15, 71–74. 10.1111/j.0963-7214.2004.01501012.x 14717835

[B43] Watson D. Clark L. A. Tellegen A. (1988). Development and validation of brief measures of positive and negative affect: the PANAS scales. J. Pers. Soc. Psychol. 54, 1063–1070. 3397865 10.1037//0022-3514.54.6.1063

[B44] Wiltermuth S. S. Heath C. (2009). Synchrony and cooperation. Psychol. Sci. 20, 1–5. 10.1111/j.1467-9280.2008.02253.x 19152536

